# Sensing Human Activity: GPS Tracking

**DOI:** 10.3390/s90403033

**Published:** 2009-04-24

**Authors:** Stefan van der Spek, Jeroen van Schaick, Peter de Bois, Remco de Haan

**Affiliations:** 1 Department of Urbanism, Faculty of Architecture, Delft University of Technology, Julianalaan 132-134, 2628 BL, DELFT, the Netherlands; 2 Chair MISSU, Multifunctional (sustainable) Spatial Use, University of Applied Sciences, PO Box 1025, 1000 BA, Amsterdam, the Netherlands E-Mails: j.vanschaick@tudelft.nl (J.S.); p.g.debois@tudelft.nl (P.B.); a.r.dehaan@tudelft.nl (R.H.)

**Keywords:** GPS, Tracking, People, Behaviour, Mapping, Movement

## Abstract

The enhancement of GPS technology enables the use of GPS devices not only as navigation and orientation tools, but also as instruments used to capture travelled routes: as sensors that measure activity on a city scale or the regional scale. TU Delft developed a process and database architecture for collecting data on pedestrian movement in three European city centres, Norwich, Rouen and Koblenz, and in another experiment for collecting activity data of 13 families in Almere (The Netherlands) for one week. The question posed in this paper is: what is the value of GPS as ‘sensor technology’ measuring activities of people? The conclusion is that GPS offers a widely useable instrument to collect invaluable spatial-temporal data on different scales and in different settings adding new layers of knowledge to urban studies, but the use of GPS-technology and deployment of GPS-devices still offers significant challenges for future research.

## 1. Introduction

### 1.1. The Global Navigation Satellite System

The availability of so-called geopositioning devices such as GPS (Global Positioning System) devices has grown enormously in the last decade and is still increasing. More and more people own a *navigation system* such as a TomTom, a *GPS for orientation* for outdoor uses, biking and geo-caching or a *mobile phone* or other *handheld communication device with built-in GPS*. These devices are mainly used for orientation (determining where you are), navigation (determining where to go) and communication (exchanging information with others or accessing information services). But the devices can also be used for *tracking*, i.e. saving a travelled route into a track log. This ability makes the technology useful to collect spatial-temporal data and thus as ‘sensors’ for observing and measuring activities of people [1].

GPS is a Global Navigation Satellite System (GNSS). GNSS is a system for location or position determination – so called geopositioning [2]. Using a special receiver, a geoposition in space and time can be calculated based on the reception of satellite signals. The United States’ Global Positioning System (GPS) was the first available system using satellite Position Determination Technology (PDT) [2]. Other GNSSs are under development in Europe (Galileo) and Russia (Glonass).

GNSS is an essential Positioning Determination Technology for many fields of study. Although in recent years the system has undergone a significant ‘modernisation’ to improve its quality [2], the capability for geopositioning in the built-up (urban) environment is still one of its major weaknesses. In particular the availability of accurate indoor signals and with low speeds is limited. The future availability of Galileo is expected to increase the performance of GPS significantly [3]. New technology such as high-sensitive GPS receivers will improve the accessibility to GPS signals.

### 1.2. Seeing the Global Navigation Satellite System as Sensor Technology

The most general definition of a sensor is ‘a device which detects or measures a physical property’ (Compact Oxford English Dictionary). A more specific definition of a sensor is ‘a device that responds to a physical stimulus (as heat, light, sound, pressure, magnetism, or a particular motion) and transmits a resulting impulse (as for measurement or operating a control)’ (Merrian Webster). Seeing GPS as a sensor requires use of the latter definition. Rather than a physical property it measures the location and motion of a GPS device and the person or object that carries it. The output is in the form of a track log with 3-dimensional spatial location coordinates and a time stamp. In an effort to characterize different types of sensors, Michahelles and Schiele [4] distinguish six sensing dimensions of which location, activity and interaction apply to GPS. Their analysis of sensor applications shows the appropriateness of placing GPS devices on both humans and objects. The experiments in this paper are limited to GPS devices carried by humans and the ‘location’ and ‘activity’ dimensions of sensing.

GPS is not the only technology for sensing location and activity of humans. For a few years already, mobile phone technology, RFID and Bluetooth can also provide easy-to-handle sensing data on location and activity. However, these technologies have their particular drawbacks with regards to the type of experiments described below. For more on those technologies see Shoval’s ideas about ‘human sensing’ [5] and tracking pedestrians [1,6] and experiments such as those by Ratti *et al* [7] and Ahas *et al.* [8] using mobile phone data, Millonig and Gartner [9] using Bluetooth, Fu and Retscher [10] using RFID and Wayn *et al.* [11] using WiFi.

### 1.3. A Concise Overview of Literature

With the increasing use of GPS and other tracking technologies, the number of scientific publications on these technologies and their application is also increasing. The aim of this paper is to add a new dimension to the evaluation of tracking studies, namely to evaluate the sensory qualities that GPS technology offers for researching measures of urban quality, where most research focuses on other fields of expertise.

Transportation science is a major field of research in which tracking studies have developed and on which tracking research builds [12]. Janelle and Gillespie [13] see ‘trackability’ as one of four major concepts in transportation sciences to understand the impact of new technologies in research, commercial practices, policy and broader society. Transportation planning is also the field where the rekindling of Hägerstrand’s theory on time-geography [14] is best illustrated [15]. Recent examples of tracking studies in transportation sciences can be found in [16] and [17]. Studies in this field show a preference for using tracking data as input for simulation and prediction models.

Studies focussing on location based services (LBS), a relatively new field of study, form the major part of GPS technology-oriented research. In their opening article to the new Journal of Location Based Services, Raper *et al.* [2] set out to critically evaluate location based services and their potential. They conclude “that there is already a huge and sophisticated body of research on LBS. However, it is poorly integrated”. They define the following key issues for future research work:

- Envisage and embody ‘blue sky’ innovations;
- Explore user experiences and social implications outside commercial implementation;
- Tackle technical problems that lead to system developments without an early return.

In general, studies related to LBS strongly attach to the importance of data visualisation (see [18]), but geovisualisation in general is an upcoming area of interest. See for example [19]. Tracking (recording route and time) as a research technique did not arise from the advent of GPS and mobile communication technology. Hill provides an overview of early non-technological tracking studies that focused on pedestrians, of which he found the earliest examples in the 1960s [20]. Studies on pedestrians in particular demonstrate that GPS tracking needs to be connected to other research techniques to fully understand movement behaviour. Millonig and Gartner, for example, use across-method triangulation, including shadowing, interviewing and counting [9]. Shoval and Isaacson use tracking technologies for urban analysis [1]. Other fields of application are environmental health [21,22] and medicine [23–25].

Future studies are another popular field of study in which tracking technologies get attention. For example, Ahas and Ular [26] go as far as to predict a fundamental change in planning and public administration. Studies in this domain do tend to be biased towards a technological paradigm of societal change, while the use and application of tracking technologies does not depend on fully pledging to a technologically determined future. Practical, pragmatically set up applications can already carry far [27].

### 1.4. Problem Setting

In general, many studies focus on technical issues regarding GPS, rather than on the application of knowledge derived from a tracking study. If they are knowledge-oriented, studies often focus on a singularly empiricist approach, rather than on application of knowledge. There is a tendency to fill the gap between empirics and application by collecting greater and greater data sets, since ‘the more data the better you know what to do’. Although for some issues, like performing diagnostics on the level of a complete transportation system, this might be so; this argument does not always hold up. Still, handling large data sets is a significant problem that has to be dealt with in all but the smallest studies using GPS, so that automation of data processing will become ever more necessary. In addition, reduction of data complexity through data visualisation might offer complementary alternatives to data mining.

However, since the major focus in the body of literature on tracking is on progress of GPS technology, studies in the fields discussed above often lack attention to the particular problems regarding knowledge application in the field of planning [27]. If the application of knowledge is addressed, this often remains limited to modelling and predicting behaviour, which does not fully use the potential of tracking data as we show below. A particular field where this gap can be felt is urban planning and design, where a central concern is urban quality. A major determinant of urban quality is the so-called use-value of space [27]. This is the reason we focus in this paper on studies that use GPS to study aspects of urban quality. Possible indicators of urban quality in an existing environment are for example hotspots (attractions, destinations, landmarks); or their counterpart ‘black holes’ (non-attractive spaces, non-space, not part of the cognitive system).

Although the sudden interest for patterns of use in tracking studies might suggest that research into use aspects of urban quality in urban design is new, there are interesting examples available based on other research techniques and approaches. For example, Gehl *et al.* developed interesting research techniques based on observation and counting [28]. Classic in the field is the work of Kevin Lynch [29] who focused on the physical characteristics of the urban environment as an experienced landscape. But most urban analysis focuses on the physical aspects of the urban environment per se such as morphology and types of buildings. GPS tracking offers to these kinds of studies a new layer which provides insight in processes and actual movement of people. In particular it adds an important temporal dimension to research in urban design primarily focussing on spatial patterns.

Hence, it is interesting to look at the value of GPS as a research technique in the context of urban studies and in particular urban design and planning for two reasons: (1) it might add to the knowledge on the use value of urban spaces, (2) it offers an intriguing new technique which enables research possibilities not available with either traditional urban analysis techniques or low-tech research techniques for studying behaviour patterns in time and space. So the main question posed in this paper is: what is the value of GPS as ‘sensor technology’ measuring activities of people, in particular in the context of studies regarding urban quality? By looking at two different research projects by TU Delft using GPS, this question is answered along three lines. Firstly, what is the contribution of GPS to traditional methods of urban research? Secondly, what is the performance of GPS as sensor instrument? Thirdly, in what ways can GPS-devices be deployed in urban settings?

In this paper we will first describe the general process and database architecture and the particular approaches used in the different case studies. Next, we show how the empirical results in the different research projects provide insight into GPS as a sensor technology with relevance for studies on urban quality.

## 2. Set up of GPS Experiments and Data Processing

### 2.1. Context of the GPS Experiments

**Figure 1.**
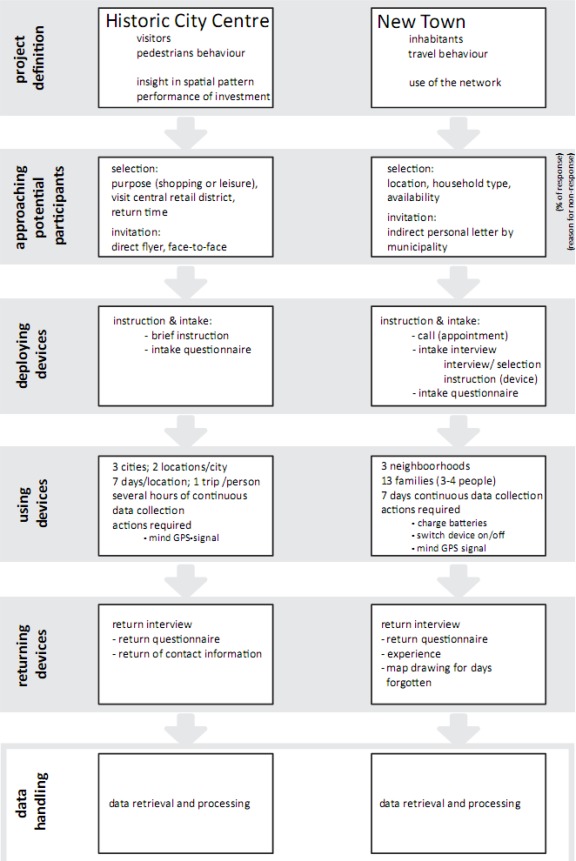
Approach to the field work.

GPS devices have been used by TU Delft to track people in several experiments. The experiments, of which we describe two here, had different research aims and took place on different scales. In the first experiment described in this paper, GPS devices were deployed in the INTERREG IIIB Spatial Metro project to observe pedestrians visiting the historic city centres of Norwich (U.K.), Rouen (France) and Koblenz (Germany) [30,31]. In the second experiment described below, the technology was used to track the activity patterns of families in Almere (The Netherlands) [32]. In both cases the collection of spatial-temporal data took one whole week and was accompanied by a questionnaire, although there were some important differences in set-up of the field work (see Figure 1). The details of both experiments’ set up are explained below in Sections 2.2 and 2.3.

The GPS-experiments described in this paper were set up to tackle questions about urban quality in the field of urbanism, i.e. the design and planning of urban areas. However, the application of data from GPS experiments in urbanism is not a matter of course. The expert meeting ‘Urbanism on Track’ in 2007 showed that GPS does not automatically bridge what can be called the *applicability gap* between empirical studies on behaviour and the making of an urban design [27]. Klaasen [33] lists the following potential problems, amounting to the *applicability gap*: (a) the tendency to collect more knowledge on restricted parts of situations, burdening the synthesising capacities of designers, (b) knowledge generated through empirical research not being geared towards the information need of designers – for example empirical researchers tending to communicate verbally, while designers tend to communicate visually, and (c) designers not formulating synthesis-oriented research questions for spatial scientists.

Despite these limitations, GPS is an interesting new instrument to map and measure urban quality in new ways. The particular cases in this paper refer to different types of urban quality that can be studied using GPS. Spatial Metro is a research program which focuses on urban quality from the perspective of pedestrians. The aim of the program is to find new and exciting ways of improving city centres for pedestrians. GPS was used as an instrument to analyse both individual routes (so called trajectories or tracks [2]) and collective, aggregate patterns of use. In addition to the Spatial Metro program, TU Delft carries out research on new towns, i.e. cities, towns, or communities that were carefully planned from its inception and are typically constructed in a previously undeveloped area. Rather than on pedestrians only, this case focused on week-long activity patterns of families and in particular on how sensing these activities gives a diagnosis of the structural quality of the framework of streets, roads and paths in new towns. In the case described in this paper, it was important to look for the discrepancy between the top down planned urban frame and the individual spatial circuits of everyday use, related to the time-space budget of individuals and families.

### 2.2. General Set up and Process Architecture

In general, the GPS experiments developed by TU Delft contain two main parts. Firstly, spatial-temporal data and background information is collected during field work. Secondly, the collected data is retrieved and processed.

The field work consists of the following four phases (see Figure 1): (a) preparation of the experiment, (b) deployment of the devices, (c) use of the devices by participants and (d) the return of the devices. The preparation of the experiment is an essential phase to determine the goals and to invite and select potential participants. Clear communication with potential participants is essential to get into contact with people and to persuade people to participate. During the deployment phase two protocols are important. On the one hand, the participants are instructed to use the protocol on the use of the devices, e.g. charging, switching on/off, waiting for position fixation. On the other hand, it is important for research assistants to use a protocol for deployment and instruction, since at deployment (as well as on returning the device) the participants get interviewed using questionnaires and because data quality also depends on proper use of the device. In the following phase, the devices are carried by the participants during the period set for the experiment. In this phase, the spatial-temporal data is being collected. The devices are a ‘black box’ for the participants that stores the data without participants being able to use all interactive functions of the device, both to reduce the burden for participants and to diminish the influence of carrying the device on the behaviour to be observed. In the final phase of the field work, the devices are returned and a post-hoc questionnaire is taken.

**Figure 2.**
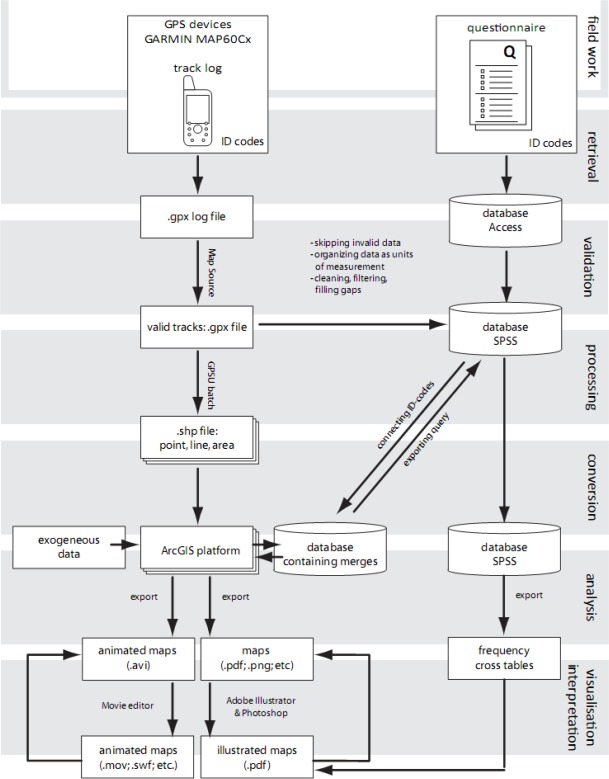
General data processing scheme for GPS experiments.

Following the field work the data is retrieved from the GPS devices and processed for further analysis. Figure 2 summarises the steps in the process. The processing of the data eventually leads to analytical drawings and maps that visualise the conclusions drawn from the data collection. In the first phase of data handling, the data is retrieved from the devices and from the questionnaires into, respectively, track log files and an Access database. The track logs contain the trajectories. After retrieval, the validity and integrity of the data is checked and both track logs and database are cleaned and filtered. After the validation process, only the valid data is used for spatial and temporal analysis.

During the processing phase the valid trajectories are converted into three types of ‘shape files’ (.shp: point, line and area) using GPSU batch utility. These shape files can be linked in ArcGIS using the Data Interoperability Tool (DIT). In ArcGIS the layers containing the trajectories are merged and connected to the Access database using a unique trip ID.

The Access database containing the interview data and some characteristics of the trips are converted into SPSS for frequency, cross-table and cluster analysis. In ArcGIS, several actions are undertaken: data is selected (query) based on the combination of SPSS and GIS and analysed using filtering, layering and calculations, such as density analysis. A distinction between two types of density calculations can be made: point density (time corrected) representing the total time spent on a location and line density representing the number of people.

In the ‘visualisation & interpretation’ phase the results of the analysis phase are exported to static and dynamic file formats leading to illustrated temporal maps (e.g. .ai or .pdf) and animated temporal maps (e.g. .mov or .swf). Based on interpretation of the results, conclusion maps are drawn. The final versions of these maps are produced in an iterative process by exporting new output from ArcGIS.

### 2.3. Approach in the Spatial Metro Cases

**Figure 3.**
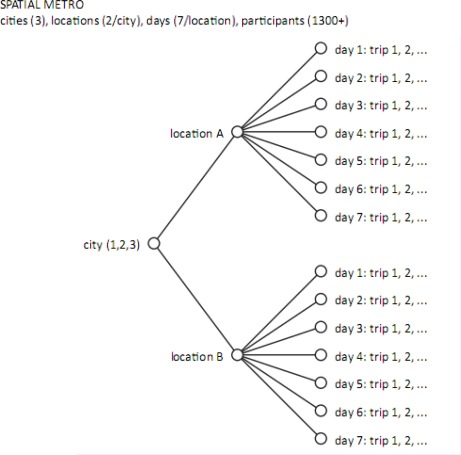
Principle of deployment of GPS devices in the Spatial Metro case. Each trip contains the complete, walked pattern of that day for one participant and is delimited by leaving the car park (location A or B) and returning to it.

For the Spatial Metro project the GPS tracking devices were distributed continuously during daytime over a period of one week at two parking facilities in each of the three cities [30,31]. These locations form the access points to the city centre for many people and is the location where these people start their journey on foot. People were asked to participate in the experiment when leaving the car park to enter the city. A flyer was used to explain the project, the experiment, the way of working and how privacy issues were dealt with. An operative GPS device was handed over to the participant to record their ‘trip’ (see Figure 3). The burden for the participant was limited to incidental check of the reception of a proper satellite signal, a ‘fix’. In the Spatial Metro project the GARMIN MAP60Cx GPS receiver was used. This is a 20-channel high-sensitive receiver based on Sirfstar III hardware. The GPS device logged position information at a frequency of five seconds. The track log was stored in the internal memory (10,000 waypoints) and automatically saved to a .gpx-file on the internal MicroSD card.

A questionnaire was filled out on return at the access point. No sensitive, private information revealing the identity of the participant was collected. Data collection was limited to trip-related data and general characteristics of the visitors. Data collection was limited to daytime use. The distribution and collection locations opened at 10 am and closed at 6 pm. Tracking data that was stored on the GPS device was extracted in office at a later stage.

In the next phase the spatial-temporal data was processed by evaluating, cleaning and validating the raw data. After that, the valid tracking data layers were projected in a Geographic Information System. In the following phase two types of drawings were made. The first type of drawings combines spatial-temporal data with e.g. an aerial image, access routes and arrival points, commercial activities, points of interest and investments. These drawings result in a visual analysis of the local spatial conditions in relation to actual behaviour of the tracked sample. The first type based on projection of the data on top of other geographical information and the second one based on density analysis of the data determined by the themes of the questionnaire.

The second type of drawings delivers a set of specific spatial patterns based on the aspects of *origin*, *familiarity*, *purpose* and *duration* [30,31] and *age*, *gender* and *group type*. These maps offer the possibility to visually compare spatial patterns for specific subgroups of participants.

### 2.4. Approach in the Case of Almere

**Figure 4.**
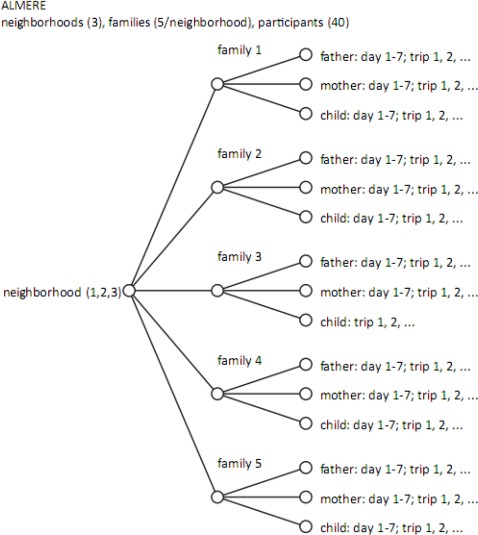
Principle of deployment of GPS devices in the Almere case. Each trip is delimited by leaving the home ‘base’ and by returning to it.

After a request from TU Delft, the municipality approached families in three neighbourhoods of the new town of Almere. Each family asked to participate in the research consisted of a father, a mother and one or two children in the age range of 16–18. This family constellation was chosen for the expected wide range of activity and movement patterns within one family unit. Initially, fifteen families agreed to participate. In the end, 40 participants of 13 families carried GPS devices for one week. The field work (see Figure 1) started with a meeting at the participant’s home to fill in a questionnaire, to give instructions about carrying the device and to hand over the GPS devices - one for each member of the family - and battery chargers (see Figure 4).

During the week of data collection all tracking data was stored in the memory of the GPS device. The spatial-temporal data was stored at an interval of two seconds. On the eighth day after distribution, the devices were collected again during which a second interview took place and maps were drawn filling in blanks when devices were forgotten by participants. Hereafter, processing of both spatial-temporal and personal data started. The first step of processing was evaluation and cleaning. The second step was to split up tracks into trips based on each round trip from home. The third step was to enter the data in a database according to the developed protocol (see Figure 2). Once the data was entered, a range of layers in GIS could be selected and connected to the Access database and vice versa to answer queries, to perform analyses, e.g. point or line density analysis in GIS or to do other quantitative analysis using SPSS.

In Almere each member of 13 families was tracked for one week, covering several hundred trips. The data provides information about use of the network over periods of 24 hours, including starting and end time and length of a trip or activity, speed and transport modes of transportation, the radius from home or from activity locations. As expected, data collection encountered several problems that led to incomplete data: a lag in time to first fixation (fix) after leaving home, insufficient charging of batteries, forgetting or switching off GPS devices by participants. But a significant set of valid data remained for visual and quantitative analysis.

## 3. Study Areas and Empirical Results

### 3.1. The Value of GPS as Sensor Technology

In this section we show how the theory behind and the empirical results from the different research projects provide insight into the value of GPS as a sensor technology in urban studies. For both cases, the particular problem setting is described, followed by an explanation of analytical methods used in the context of that problem and how GPS tracking fits. Both case descriptions provide insight in the main measurements to be performed on GPS data to be relevant for that particular problem setting.

### 3.2. Spatial Metro: Norwich, Rouen, Koblenz

Norwich, Rouen and Koblenz are relatively small cities (100,000–130,000 inhabitants) located in the United Kingdom, France and Germany, respectively. Each city has a historical centre and functions as a regional attractor serving a large hinterland. Concerned about future retail developments, the administrations of these cities struggle to keep up the vitality of their urban core as the central shopping district and in particular as an attractive place for living.

The aim of the overall Spatial Metro project is to make investments in public space happen; in particular to improve the city centres for pedestrians. In this light, the role of TU Delft was to develop tools to measure the effects of the investments in for example city beautification, street furniture, lighting and information systems [30]. Based on the outcomes of the studies, further plans and strategies to improve the city centres for pedestrians were drawn.

TU Delft developed and used two tools: (1) street interviews to collect information about the experiences of visitors (used in 2005 and 2006) and tracking technologies (GPS tracking) to collect data on actual movement and routing (used in 2007). Sixty-six people were interviewed in 2005. The interviews resulted in maps of great public spaces: ‘great to shop’, ‘great to enjoy’, ‘great to discover’ and ‘the greatest’. Further it resulted in an indication of gender, frequency of visit, age, origin, time spent, money spent, access mode and purpose.

Using the GPS devices, in total 1,300 pedestrians were tracked and interviewed. On average 60% of the data was valid. The remaining 40% was not usable due to problems with fixation, batteries, blur (clouds of points) and fragmentation. The origin of these issues is clear: the signal reception in dense urban areas is weak; signals reflect on buildings, people tend to go into buildings, and pedestrians move relatively slowly (see also [2]). Also, the data collected using GPS must be seen as only representing the behaviour of visitors starting from those specific access points where GPS devices were distributed. Nevertheless, a substantial amount of data remained to map significantly differing and meaningful spatial patterns based on the social/demographical characteristics of participants and based on trip aspects.

Most insight gained from the experiment came from the spatial-temporal data adding another layer of information to the interview data and to the spatial analysis, providing in particular more insight in daily processes in the city. The combination with information exogenous to the GPS data, such as morphological maps, provides a technique to discover anomalies and to draw conclusions about qualities of public space. Density analyses offer a tool to discover ‘hotspots’ of use in the city based on actual movement, not on perception and post-hoc questionnaires. The use of a questionnaire offers a method to distinguish between trajectories on several different aspects, providing unique thematic maps and the ability to compare different aspects based on SPSS cross-tabulations. Finally, the use of dynamic maps for data visualisation offers temporal diagrams. These show not only trajectories, but also directions and flows of movement.

Both static and dynamic ways of visualisation offer tremendous insight in pedestrian behaviour, leading to conclusions about differences in urban quality between places and about opportunities for improving the local situation, which can be applied in planning practice.

To get the full picture, data collected using GPS should be used in combination with data on the location of programs, activities and points of interest, morphology and quality of public space, and with counting data. Placing counting devices in the right locations might provide a system to extrapolate the GPS sensor data.

### 3.3. Diagnostics of New Towns: Almere

New towns have a number of generic characteristics. The plan to develop a new town is generally derived from an unambiguous socio-economic paradigm. The spatial layout and stratification of the circulating system, using subsystems of different speeds, is based on avoiding risk and conflict between transport modes and inhabitants. Most new towns lie within the influence sphere of an old, central city and often depend on it functionally. The plan for a new town often projects a particular life style, which projection is supposed to solve the social and spatial problems of the dominant central city. In general, new towns are built up in a short time with a simple or particular physical structure. The construction of housing in new towns conforms to market principles and relies on a fast and efficient building process for large groups of inhabitants that mostly originate from the central, dominant city. Almere is a morphologically polynuclear new town in the reclaimed land of the Flevopolder. The town exists of spatially separated suburban neighbourhoods. New towns in general and Almere in particular has attracted heavy criticism in the last decades with regard to mobility issues and quality of living, the need for renewal of some its parts, the (re)development of a city centre and the continuing planning tasks of housing construction.

Over the last decade TU Delft developed and applied several spatial-analytical methods [34–37] to analyse the functioning of the city and the relation between the different parts. The resulting theory of functional-spatial structure is the basis for the GPS study on Almere described in this paper. Firstly, this theory explains how a city is constituted of (a) ‘frame’, i.e. the network of highways, roads, streets and paths, (b) ‘pattern’, i.e. the spatial distribution of programs and place qualities in a city, and (c) ‘circuit’, i.e. the paths people take to get around the city. It works from the assumption that a complete and healthy frame compiles flows and social encounters into meaningful and productive public spaces [35]. The structure of the urban frame of a city is a precondition for the way users of public space of the city have access to the city as a whole and to the interconnected neighbourhoods. It facilitates the circuits of movement in the city related to retail centres and urban ‘anchor points’ like parks and other places of activity in the urban landscape. The theory states that the completeness of the urban frame is in fact a precondition for the identity and liveliness of the city as a whole.

**Figure 6.**
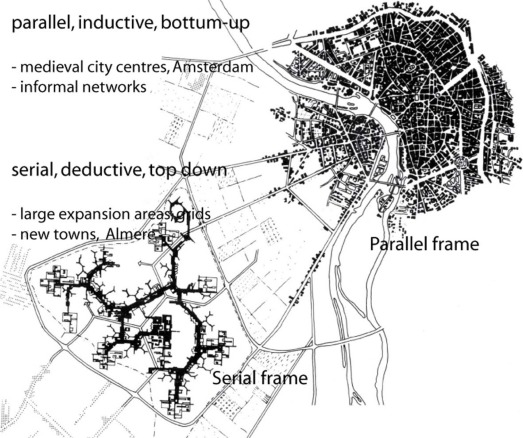
The principal difference between a ‘parallel’ and ‘serial’ urban system.

Secondly, this theory explains how the process of planning and construction of cities can be related to the physical structure of the urban frame. In particular, the factor time in the process of planning has a strong influence on the structure of public space, i.e. the structure of the urban frame and of parks and squares in the city. The theory distinguishes between urban systems that are organized as a ‘parallel’ system or as a ‘serial’ system [34,38] (see Figure 6). The planning discourse during the period of the first new town developments dictated the planning of cities as a ‘serial’ process of making decisions. Therefore, in contrast to the ‘parallel’ process of long-term development in ‘grown’ cities, the initial set-up of rationally planned, rapidly developed new towns is generally ‘serial’ in nature. And as a consequence, new towns have become cities with a ‘serial’ spatial urban frame. This second part of the theory, states that a truly *urban* life needs a ‘parallel’ organised urban frame.

So far, methods of analysis in Almere have focused on analysing the spatial structure, layout and process of planning. The GPS tracking experiment, part of ongoing studies on Almere, produces a new layer adding a mapping of actual behaviour to this analysis. This could contribute to substantiating or falsifying the theory on ‘parallel’ and ‘serial’ systems constructed of ‘frame’, ‘pattern’ and ‘circuit’.

**Figure 7.**
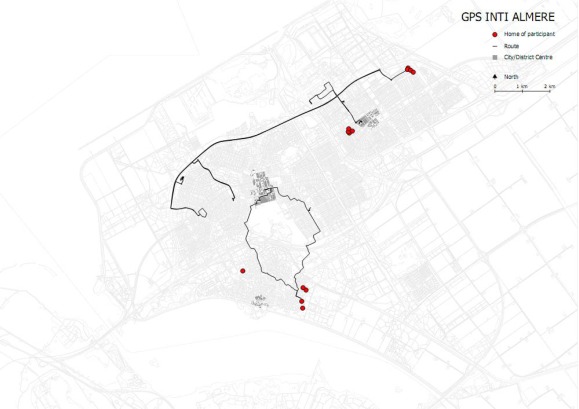
Two ‘extreme’ types of GPS tracks from the Almere experiment: degree of match between both ways on a return trip. Background map is based on the Almere street pattern.

The problem for which GPS is used in the example of Almere is the following. In a ‘serial’ spatial urban frame there is often only one way to connect points of origin and destination, to connect the sequence of activities during a day. But, quality of life and the accommodation of multiple life styles demand a wider range of choice of possible routes, differing in time and space. The quality of urban life derives from coincidences, i.e. of ‘parallel’ use of the spatial urban frame, a condition for being related with others and other places in time and space. One possible measure of urban quality then is the degree to which round trips use the same route back and forth (see Figure 7).

Another interesting measure made possible by tracking research is accumulated length of trip for particular purposes. The GPS tracking project in Almere made clear that in spite of an extensive pedestrian network and cycling network, parents transport their children to sport facilities during the week largely by car. Preliminary data analysis and extrapolation suggests that this might add up to a staggering total weekly amount of 800,000 to 1,200,000 km just for the city of Almere [32]. Such data is hard to get through other research approaches, while such measures can be relatively easily deducted from GPS data. This gives rise to the development of new hypotheses in urban studies on new towns, such as the possibility of cognitive dissonance with regard to the choice for transport by car.

**Figure 8.**
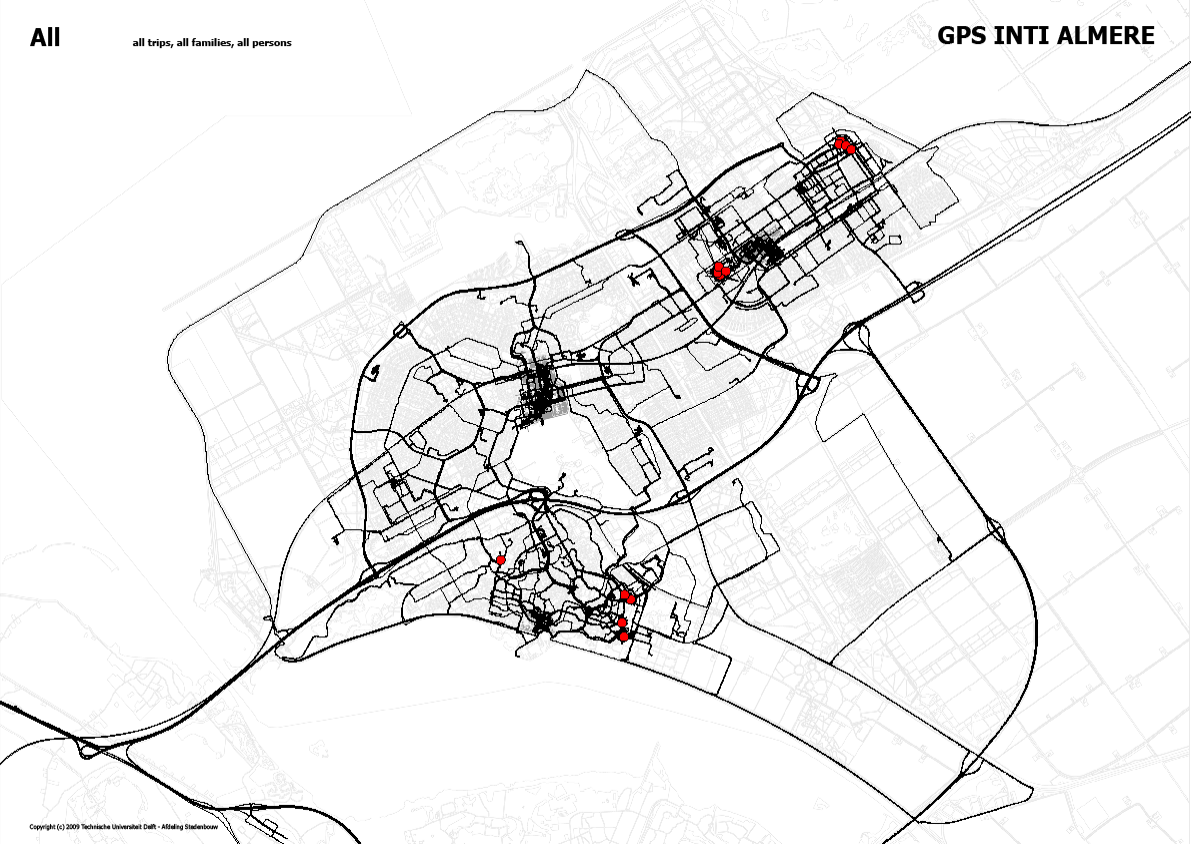
GPS tracking data for one week of the Almere experiment: superimposition of activity patterns of 13 households (40 people), red dots indicate housing location. Background based on the Almere street pattern.

In the case of Almere, there seems to be a partial paradox between the ‘serial’ process of planning the town resulting in a hierarchical structure of the urban frame and the crisscross way in which people use this urban frame by living in it day and night (see Figure 8). However, initial results of the tracking experiment also seem to show that the ‘serial’ frame limits inhabitants considerably in their choices for moving around town. While mapping traces of use is fascinating by and for itself, making it possible to see in maps how people really use the available network is extremely important for communicating about quality of urban life in relation to possible future developments with stakeholders in planning, including politicians.

## 4. Discussion

TU Delft used GPS tracking technologies in two experiments on different scales and in different ways of device distribution. In this paper we aimed to assess the value of GPS as a sensor technology. The two different research projects described in this paper provide some insight there. The main question in this paper was: what is the value of GPS as ‘sensor technology’ measuring activities of people, in particular in the context of studies regarding urban quality? By looking at two different research projects by TU Delft using GPS, this question is answered in this section along three lines. Firstly, what is the contribution of GPS to traditional methods of urban research? Secondly, what is the performance of GPS as sensor instrument? Thirdly, in what ways can GPS-devices be deployed in urban settings?

Traditional methods of research in urban design and planning are mostly visual in nature, such as morphological analysis, functional-spatial analysis, exploration of design alternatives, superimposition of thematic maps, etcetera. Although the interest in actual use of, in particular, public space has been present since the birth of the profession of urban planning, the actual use of methods studying it have remained limited. In the field of geography, the study of temporal-spatial patterns of use has developed, in particular in the field of time geography [14], but has largely failed to translate this into applicable knowledge for urban design and planning. One reason for that is the quantitative, rather than visual, nature of most studies in that field, but also the lack of accuracy in measuring variables relevant to the design of public space and the structure of public spaces in cities might have played a role. Another factor is the difficulty of bringing together many different strands of information in urban design and planning as required for solving the type of problems akin to the field.

So, the contribution of GPS to traditional methods of urban research can be valued from these three angles: visualisation, accuracy and the validation of other research techniques. Visualisation plays a very important role in dealing with tracking data. In the processing stage it is important in case of manual validation of data. In the interpretation and analysis stage it is important as a tool for analysis. And last, but not least, visualisation is instrumental in communicating research results from tracking studies both to experts across disciplinary boundaries and to non-experts such as politicians or civilians. The visual nature of tracking information makes it more akin to other research techniques in urban planning and design than previous research techniques to analyse people’s behaviour patterns in space and time. So, on the one hand the easily visualisable tracking data connects directly to the visual data used in urban planning and design – it can be literally layered on top of each other - while on the other hand it brings previously numerically and verbally oriented fields of knowledge into the language domain of urban planning and design. With regard to accuracy, GPS is proving to be significantly more accurate for registering routes and activity locations than post-hoc mapping or diary taking by participants. Moreover, research based on GPS tracking, and possibly in the future complete ‘urban sensor systems’, gives not only the possibility to visualise real-time behaviour. More important, it assists professionals in understanding and validating the performance of traditional urban analyses.

Looking at these three angles on the value of GPS in relation to other methods of urban research, most important is that this knowledge provides new – visual, accurate and combinable - arguments and as such a new evidence base for projecting and predicting future urban developments.

The performance of GPS as sensor instrument depends on a number of factors, both related to the technology and to factors outside the technological features. We highlight the importance of technological improvements, the scale of tracking studies, and the role of the experiment protocols.

Technically and practically GPS tracking still has its problems and for research purposes further technological development is necessary. Increased quality of GPS-receivers and, for example, integration with other technologies such as the mobile phone system, is important to improve the quality of spatial-temporal data collection. Important technical issues are battery life time, time to (first) fix, accuracy of reception and avoiding blur and fragments. In addition to hardware improvements, software - such as scripts and data-mining algorithms - needs to be improved to further raise the quality and to speed up data processing.

The experiments confirm the findings in Schaick and Spek [12] that scale limitations are a determining factor in the set up of tracking experiments. Three types of scale and the relations between them are important: (a) spatial delimitation of the experiment, (b) temporal delimitation of the experiment and (c) the number of participants. For example in the Spatial Metro case, it is a matter of concern that the total number of participants leads to a small number of trajectories per subgroup. Practically, the distribution and collection of devices limited the deployment of the technology to a pre-defined environment since this process is time consuming and limits the type of participants. The experiments did not allow for compensating for so-called ‘observer effects’ [9].

The cases show that data quality is not only dependent on the technical performance of the global positioning system. In the set up described in this paper, data quality very much relies on the parties involved. More than in the case of Spatial Metro, data quality in the Almere case is dependent on the involvement of the participants, as well as their commitment to and understanding of what they are doing. In comparison, data quality in the Spatial Metro case was heavily dependent on commitment and understanding of the experiments by research assistants. In the former the use protocols were most important, while in the latter the distribution protocol was essential to uphold data quality in the end, both with regard to technical issues as well as diminishing the risk of unusual routes taken by participants as a result of participating in the experiment. The extended time period in the Almere case enabled further support during the experiment such as a manual, a quick user guide and a 24/7 service desk).

Our third criterion for valuing GPS as sensor technology is the possibilities it offers for deploying GPS-devices in urban settings. Technically, the experiments show – confirming the literature - GPS is not ideal in dense urban environments, for example because of loss of signal and reflections of the surfaces in urban environments. Furthermore, data completeness and accuracy suffers from people going in and out of buildings. Since GPS experiments are still dependent on distributing GPS devices that are carried by people or vehicles, urban environments do offer places such as car parks to control distribution of devices in a controlled setting, but the logistics of experiments are a limiting factor. Although these issues lead to a decreased amount of possibilities for carrying out tracking experiments, data validation still leaves a good portion of data. However, due the type of errors caused by deployments in urban settings, data processing in the experiments has proven to be a time consuming process.

In general we conclude that GPS offers a widely useable instrument to collect invaluable spatial-temporal data on different scales and in different settings adding new layers of knowledge to urban studies, but the use of GPS-technology and deployment of GPS-devices still offers significant challenges for future research. In both cases the GPS delivered spatial-temporal data next to background information gathered by a questionnaire. The spatial-temporal data delivered a new layer of information on top of other geographical information. The strong feature of the method is the combination of spatial-temporal data (behaviour in space and time), spatial conditions, social-demographic information of the participants and characteristics of the trips. This enables to split up patterns based on personal and trip related aspects. The methods, as developed by TU Delft, add information to the traditional ways of analysis and do therefore not a priori replace existing ways of collecting information, such as counting and observing. Still, the current experiments only show a small range of applications in comparison to what is possible, in particular to further study the qualities of public space in particular and of urban systems in general.

## 5. Conclusions and Outlook

To conclude this paper we would like to indicate a number of future developments in light of the experiments by TU Delft. We want to highlight the following developments: (1) the almost ubiquitous availability of geopositioning data in a nearby future, (2) the combination of positioning sensors with other types of sensors, (3) automation of data collection and processing, (4) the evaluative capacities of tracking technologies, and (5) the increased relevance of multiple, visual environments to communicate results from tracking studies.

Firstly, the above section showed that the scale of experiments is limited considerably because of logistical and data processing concerns when using the current generation of available GPS devices. If GPS would be available wider and people could just share their tracks, the only thing needed is a platform and a set of protocols to upload spatial-temporal data. This would enable open-source collection of spatial-temporal data and therefore feature GPS as a worldwide applicable sensor technique collecting spatial-temporal data, quantitative information and qualitative information.

Secondly, we foresee that combining GPS with other types of sensors, such as those measuring air quality, and sensory and emotional experiences of people would enable a next generation of tracking research to offer more directly applicable knowledge to the field of urban planning and design. This would require the development of new process architectures that combine several different data sets and the connection of data flows from stationary and mobile sensors.

Thirdly, automatic recognition of individual track aspects is necessary to enlarge the scale of tracking experiments. Important variables with regard to individual tracks for which automation of data processing might be valuable are: (a) destination (e.g. location, duration, activity), (b) route (e.g. track type (spatial pattern), direction), (c) exit and access and direction with regard to a delimited research locale. With regard to the automatic recognition of collective, aggregate variables would be relevant: (a) flows of masses of people and (b) emerging shared destinations (e.g. ‘hot spots’, activity, duration).

Fourth, experts in tracking research and urban planners interested in these instruments foresee that GPS tracking can be used as a tool for evaluating the effects of interventions, i.e. the transformation of urban areas, or for continuous monitoring instrument of urban areas [12]. However, since on the one hand this requires a longer term commitment to a tracking study from involved parties this, and on the other hand tracking technologies are relatively new, this type of study has not been set up yet.

Lastly, in addition to the need for increasingly sophisticated data handling on the quantitative side of GPS-data analysis, visualisation remains in our view at the heart of the strength and relevance of using tracking technologies in urban studies. With this outlook, we see tracking studies evolving from the experimental stage to a full blown research instruments in a wide range of knowledge domains.

## Figures and Tables

**Figure 5a:**
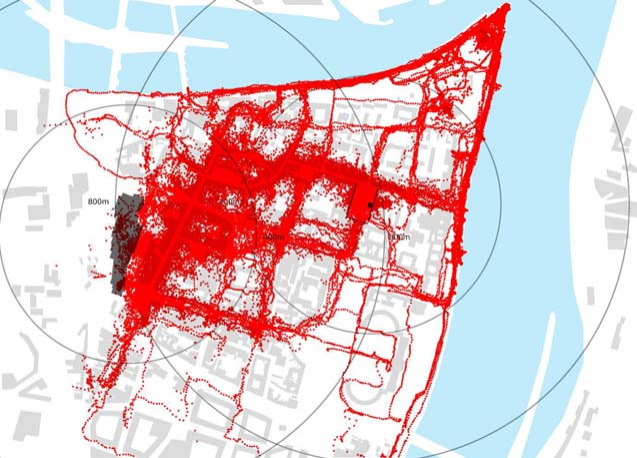
GPS tracking results from Löhrcenter (parking for 1,400 cars) and from Gorresplatz (parking for 386 cars) in Koblenz: superimposition of one week of data collection from both locations. All track points are logged at 5 seconds frequency on devices carried by pedestrians that on the same day access the city centre from the car park and return to their car.

**Figure 5b:**
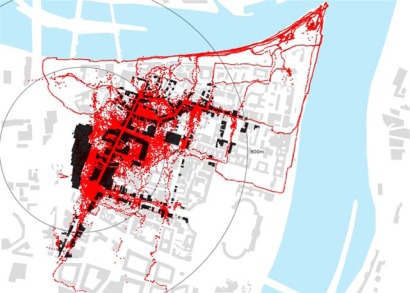
GPS tracking results from Löhrcenter in Koblenz (parking for 1,400 cars) superimposed on location of commercial functions.

**Figure 5c:**
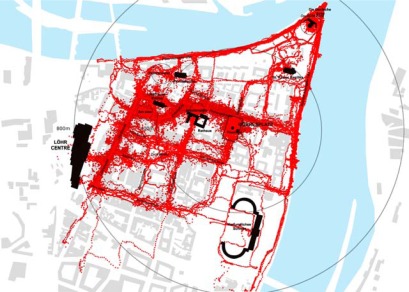
GPS tracking results from Gorresplatz in Koblenz (parking 386 cars) superimposed on location of touristic attractions
